# Neutrophils to lymphocytes ratio between benign and malignant thyroid nodule

**DOI:** 10.12669/pjms.37.7.4503

**Published:** 2021

**Authors:** Nazia Haider, Zahid Mahmood, Fizzah Khalid, Saad Abdul Razzak

**Affiliations:** 1 Dr. Nazia Haider (MBBS, FCPS General Surgery Resident), Jinnah Postgraduate Medical Centre, Karachi, Pakistan; 2 Dr. Zahid Mahmood (FCPS, FRCS, FACS), Associate Professor, Jinnah Postgraduate Medical Centre, Karachi, Pakistan; 3 Dr. Fizzah Khalid (MBBS, FCPS General Surgery), Jinnah Postgraduate Medical Centre, Karachi, Pakistan; 4 Dr. Saad Abdul Razzak (MBBS, FCPS General Surgery Resident), Jinnah Postgraduate Medical Centre, Karachi, Pakistan

**Keywords:** Thyroid nodules, Neutrophils to lymphocytes ratio

## Abstract

**Objectives:**

To determine the relationship of Neutrophils to lymphocytes ratio (NLR) values with the benign and malignant thyroid nodules.

**Methods:**

In this cross-sectional clinical study conducted from September 1^st^ 2020 to February 28^th^ 2021, we included 216 patients who underwent thyroidectomy at Jinnah Postgraduate Medical Centre Karachi Pakistan. After thyroidectomy specimens were sent for pathologic examination. Patients were divided into two categories based on histopathologic results; Malignant nodule and benign nodules. Data of complete blood count was obtained from the pre-operative lab investigations and NLR was calculated.

**Results:**

There were 42 (26%) men, and 116 (74%) women of 158 in the BTN group, 18 (31%) men, and 40 (69%) women of 58 in the MTN group. The mean age of 48 ± 6 years in the BTN group as well as 47 ±8 years in the MTN group (p-value 0.32). The mean neutrophil count in the BTN group was 4.26 ± 2.8 versus 4.41 ± 2.2 (x 1000/mm^3^)) the malignance thyroid group (p-value = 0.71). The mean lymphocyte count was 3.81 ± 0.9(x 1000/mm^3^) in the BTN group and 3.61± 1.2 (x 1000/mm^3^) in the malignance group (p-value = 0.18). The mean NLR value for the benign thyroid nodular group was 1.19 ± 2.2 and 1.22±1.8 in the malignant thyroid nodular group (p-value = 0.92).

**Conclusion:**

According to the results of this study, we concluded that preoperative period biochemistry laboratory results such as neutrophils count, lymphocyte count, and NLR value don’t provide enough evidence to differentiate between benign and malignant thyroid carcinoma.

## INTRODUCTION

Iodine deficiency is one of the reasons for developing thyroid nodules, a thyroid nodule is a commonly detected condition, women are more prominent than men. About 19 % to 68% of thyroid nodules in the population are diagnosed by ultrasonography. Generally, these nodules are benign thyroid nodules, only 7% to 15% of total thyroid nodules are malignant.^1^ Thyroid carcinoma is an endocrine malignancy. Anatomically, the papillary or follicular subtype of thyroid carcinomas derives from the follicular epithelial cells.^2^ There are a smaller number of thyroiditis patients who reported thyroid carcinomas.

Leucocytes (white blood cells) are actively participating in inflammation. The increasing inflammation response develops in contrast to external and genetic factors. There is consistent evidence that genetic and external factors have a key role in the physio-pathogenesis convert to malignant thyroid carcinomas.^3^ Recently, other independent predictive factors revealed for thyroid carcinomas in humans such as neutrophil to lymphocyte ratio (NLR), C-reactive proteins, Glasgow prognostic score, interlukin-6 (IL-6), interlukin-8 (IL-8), and albumin concentration.^4^, ^5^ these inflammation markers are reliable, cost-effective, and easily assayed to evaluate the cancer prediction. NLR investigation is one of the inflammation response indicators, which is inexpensive and universally available.

Based on recent studies, inflammation was associated with prognostic thyroid carcinoma.^6^, ^7^ There is an association of high NLR and different types of carcinomas such as lung cancer, hepatic cancer and breast cancer, etc.^5^, ^8^

The aim of the study was to determine the relationship of Neutrophils to lymphocytes ratio (NLR) values with the benign and malignant thyroid nodules.

## METHODS

In this cross-sectional clinical study conducted from September 1^st^ 2020 to February 28^th^ 2021, at Jinnah Postgraduate Medical Centre Karachi Pakistan., we included 216 patients who underwent thyroidectomy Patients of age 18 and 65 years, of either gender were included. The exclusion criteria were as follows: an active infective patient, type-2 diabetes mellitus, and other inflammatory diseases, such as rheumatoid arthritis, malignancy, and pregnancy. Patients on cardio vascular disease with aspirin treatment were also excluded from the study.

Approval of the research proposal was obtained from the ethical review committee of the hospital (Ref: NO.F.2-81/2020-GENL/49285/JPMC, Dated: 2-11-2020). After thyroidectomy specimens were sent for pathologic examination. Patients were divided into two categories based on histopathologic results; 1. Malignant nodule group and 2. benign nodule group.

We collected all the pre-operative data from the biochemistry laboratory of our hospital such as hemoglobin values, white blood cell count (WBC), hemogram, hematocrit (Hct), platelet count, neutrophil count, lymphocyte count and mean corpuscular volume (MCV). The NLR was calculated as absolute neutrophil count divided by the absolute lymphocyte count.

Data were analyzed in SPSS v25 software. Quantitative variables were calculated as Mean ± SD, NLR ratio among patients with malignant and benign nodules was compared using independent sample t-test. P-value ≤0.05 was taken as significant.

## RESULTS

After thyroid nodule pathologic examination results, based on their pathological condition we categorized patients into two groups, 158 patients were diagnosed as benign thyroid nodules (BTN group), 58 patients are diagnosed as malignant thyroid nodules disease (MTN group).

There were 42 (26%) men, and 116 (74%) women of 158 in the BTN group, 18 (31%) men, and 40 (69%) women of 58 in the MTN group. The mean age of 48 ± 6 years in the BTN group as well as 47 ±8 years in the MTN group, there was no significant difference in age (p-value=0.32). The pre-operative laboratory results have no statistically significant correlation between the groups, either Hb, Hct, WBC count, MCV and platelets count, p= 0.75, p= 0.33, p=0.64, p = 0.21 and p = 0.32 respectively. The mean neutrophil count in the BTN group was less than the malignance thyroid group (4.26 ± 2.8 versus 4.41 ± 2.2 (x 1000/mm^3^)) even though, there was no statistically significant difference found between the groups (p-value = 0.71). The mean lymphocyte count was 3.81 ± 0.9(x 1000/mm^3^) in the BTN group, 3.61± 1.2 (x 1000/mm^3^) in the malignance group (p-value = 0.18), there was no statistically significant difference of neutrophil count and lymphocyte count in between the groups. The NLR values were calculated based on the formula, the mean NLR value for the benign thyroid nodular group was 1.19 ± 2.2 and 1.22±1.8 in the malignant thyroid nodular group. There was no statistically significant difference found between the groups (p-value = 0.92). (Table-I)

**Table I:** Baseline Demographic and Biochemistry Laboratory Values.

	*BTN group (n=158)*	*MTN group (n= 58)*	*p-value*
***Gender***			
Men n (%)	42 (26%)	18(31%)	0.93
Women n (%)	116 (74%)	40(69%)	0.68
Age (years)	48 ± 6	47 ±8	0.32
Hb (g/dl)	12.2 ± 2.2	12.3 ± 1.8	0.75
Hct (%)	36.8 ± 2.1	37.1 ± 2.4	0.33
WBC (x 1000/mm3)	7.3 ± 3.2	7.1±1.2	0.64
MCV (fL)	85 ± 5	84 ± 6	0.21
PLT (x 1000/mm3)	282 ± 56	291± 68	0.32
Neutrophil count (x 1000/mm3)	4.26 ± 2.8	4.41 ± 2.2	0.71
Lymphocyte count (x 1000/mm3)	3.81 ± 0.9	3.61± 1.2	0.18
NLR	1.19 ± 2.2	1.22±1.8	0.92

Hb = hemoglobin, PLT = platelets, WBC = white blood cells, Hct = hematocrit, NLR = neutrophil-lymphocyte ratio, MCV = mean corpuscular volume.

## DISCUSSION

Thyroid nodule has been directly related to age, gender, smoking habits, obesity, and metabolic disorders.^1^ Patients receiving upper-body radiotherapy are prone to increased risk of thyroid nodules. In present study, we conducted the association between the NLR and its association with benign and malignant thyroid nodules. Our study goal was to identify the biochemistry laboratory values are providing enough evidence about the existence and pathology of the nodules or not. Normally NLR values are elevated levels in patients having coronary artery diseases, hypertension, and type-2 diabetes. We excluded such patients from our study.^9^,^10^

Research publications recommend NLR is a novel inflammatory marker, it has strong relevance with cancer. malignancy itself stimulates inflammation and produce inflammatory markers.^11^ Inflammatory cells release cytokines which interact with bone marrow where lymphocyte and neutrophils are produced. There is strong evidence of systemic inflammation has a connection with neutrophilia and lymphocytopenia thus NLR may be one of the inflammatory markers.^12^,^13^ in our study there were non-significant neutrophil count and lymphocyte count has been detected in benign nodule and malignant thyroid nodule.

The debate between cancer and NLR has been taking the attention of interest. Preoperatively elevated values of NLR has prognostic values for the other type of cancers, such as ovarian cancer, colorectal cancers, gastroesophageal cancers, lung cancer, and pancreatic cancer.^14^-^16^ Gomez et al. has suggested that increased level of NLR could be associated with tumor invasion and higher chances of nodal disease and hepatic carcinoma.^17^

A pilot study conducted by the Seretis C et al. found there was an elevated levels of NLR was reported in advanced papillary thyroid carcinoma.^18^ Kim et al. also identified elevated level of NLR in benign and malignant thyroid nodules but failed to describe the basic mechanism.^19^ Liu et al. studied the relation between the 318 malignant thyroid nodule patients with benign nodules. He testified that there are elevated preoperative NLR values that correlate with tumor size.^20^

A study conducted by Yu et al. on a total of 560 thyroid carcinoma and healthy individuals and concluded there was lower mean platelet volume in malignant nodules compare to benign nodules.^21^ On other hand, Yucel E et al. conducted a retrospective study on 216 benign and malignance thyroid nodule patients, they didn’t find any difference between the platelet count, MPV, and NLR.^22^ Even, in our study, there were no elevated NLR levels found. We didn’t determine a relative association of NLR between benign thyroid nodules and malignant thyroid nodules.

### Limitation of the study

The study sample size and period were small, for more accurate results there is a need to conduct the study at a large center where thyroid nodules patients flow is high.

## CONCLUSION

According to the results of this study, we concluded that preoperative period biochemistry laboratory results such as neutrophils count, lymphocyte count, and NLR value don’t provide enough evidence to differentiate between benign and malignant thyroid carcinoma.

### Authors’ Contribution:

**NH:** Conceived, prepared the manuscript, and is responsible for originality of study.

**ZM:** designed the research methodology, supervised the research work, did review and gave final approval for publication.

**FK:** Helped in data collection and compilation and helped to finalize the manuscript.

**SAR:** Did data Analysis, did review.
